# Effect of the Addition of Naringenin Derived from Citrus on the Properties of Epoxy Resin Compositions

**DOI:** 10.3390/molecules29020512

**Published:** 2024-01-19

**Authors:** Malgorzata Latos-Brozio, Anna Masek, Leszek Czechowski, Aleksandra Jastrzębska, Sebastian Miszczak

**Affiliations:** 1 Institute of Polymer and Dye Technology, Faculty of Chemistry, Lodz University of Technology, Stefanowskiego 16, 90-537 Lodz, Poland; 2 Department of Strength of Materials, Faculty of Mechanical Engineering, Lodz University of Technology, Stefanowskiego 1/15, 90-537 Lodz, Poland; leszek.czechowski@p.lodz.pl; 3 Institute of Materials Science and Engineering, Faculty of Mechanical Engineering, Lodz University of Technology, Stefanowskiego 1/15, 90-537 Lodz, Poland; aleksandra.jastrzebska@p.lodz.pl (A.J.); sebastian.miszczak@p.lodz.pl (S.M.)

**Keywords:** citrus polyphenol, naringenin, epoxy resin, composites, solar aging

## Abstract

This research concerns the modification of commercially available epoxy resin with flame retardants in order to obtain aging-resistant and antimicrobial polymeric materials with a plant stabilizer dedicated to use in rail transport. Polymer compositions based on epoxy resin, fiberglass fabric, and naringenin were prepared. Naringenin was added as a natural stabilizer at 2, 4, and 8 phr. The materials were subjected to solar aging lasting 800 h. The hardness of the samples, surface energy, and carbonyl indexes were determined, and the color change in the composition after aging was analyzed. In addition, microscopic observations, analyses of mechanical properties, and microbiological tests were performed. The hardness determination showed that the samples retained their functional properties after solar aging. The increase in the polar component of the surface energy of all materials indicated the beginning of the degradation process of the composites. The tensile one-directional tests were carried out for plane samples taken in three directions (0, 90, and 45 degrees referred to a plate edge) before and after the aging process. The addition of naringenin did not affect the functional and surface properties of the epoxy resin-based materials. Polyphenol stabilized polymer composites, as evidenced by the results of carbonyl indexes. Moreover, the obtained samples showed good antimicrobial properties for *E. coli* and *C. albicans* in the field of testing the viability of microbial cells in contact with the tested surfaces.

## 1. Introduction

Natural additives in the technology of polymeric materials are of great interest. Due to the growing awareness of society regarding environmental protection and EU legal restrictions, interest in the use of additives of natural origin is increasing [1]. Literature reports indicated the possibility of using natural fibers as a green additive to epoxy composites [2]. Natural fibers have been shown to act as reinforcing fillers in materials based on epoxy resin [3,4]. There is also interest in using post-agricultural waste such as walnut shell [5], hazelnut shell, and sunflower husk [6] as ecological fillers for resin composites. Furthermore, bast and leaf fibers were used to modify epoxy composites [7].

The literature lacks information on the application of polyphenols as natural additives to epoxy resin composites. Plant polyphenols have anti-aging properties; therefore, they have been proposed as pro-environmental stabilizers of polymeric materials [8]. Polyphenols contain at least one phenolic hydroxyl group, which determines the antioxidant activity of these compounds. It has been shown in the literature that the main pathway of the radical scavenging effect of phenolic compounds is the transfer of a hydrogen atom from the phenolic hydroxyl group to the reacting radical [9]. Flavonoids are one of the groups of polyphenols for which the mechanism of antioxidant activity has been well described. The number, position, and type of substituents affect the ability of flavonoids to deactivate free radicals and to chelate metals. Moreover, the total amount and place of substitution of hydroxyl groups have a significant impact on the antioxidant properties of flavonoids [10,11]. Due to strong antioxidant properties, polyphenols have been proposed as plant stabilizers of polymeric materials of various classes. In polyolefins, flavonoids have been used as stabilizers for polyethylene [12,13,14,15] and polypropylene [16]. Silymarin can be used as a natural anti-aging additive to ethylene-propylene elastomer (EPM) [17]. Biodegradable polymers such as aliphatic polyesters, polylactide (PLA), and polyhydroxyalkanoates (PHA) were stabilized with hesperidin and rutin [18], as well as catechin and polydatin [19].

The literature contains several reports of attempts to use polyphenols as aging stabilizers for epoxy resins. The effects of resveratrol and pyrogallol on the physicochemical, mechanical, and biological properties of epoxy resin sealants were investigated. Polyphenols improved the mechanical properties of sealants, as well as increased antioxidant activity and antibacterial activity against *Enterococcus faecalis*. The authors concluded that the addition of specific polyphenols to the epoxy resin may be a suitable strategy to impart some antioxidant and antibacterial potential, along with improving some mechanical properties [20].

Currently, epoxy materials have potential in industrial products. New possibilities for applying modified epoxy resin to cement mortars are described [21,22]. A significant branch of the industry where epoxy resins are used is public transport, including the railway industry [23]. In modern railways, metal parts of seats have been replaced with polymer composites, including materials based on epoxy resin, which have appropriate mechanical properties [24]. Due to climatic conditions (UV radiation and variable temperatures), epoxy polymer compositions used as seats require stabilization [23,25].

In this paper, naringenin was used as an anti-aging additive in composites with epoxy resin and glass fiber. Naringenin is a 4′,5,7-trihydroxyflavanone belonging to the group of flavonoids. This polyphenol is especially found in many citrus fruits. Naringenin has been shown to have antioxidant, anti-inflammatory, and antimutagenic properties, and studies have suggested that it may be a useful chemoprotective agent [26]. According to the literature data, naringenin was tested as a natural antioxidant and UV light stabilizer for polypropylene [16], as well as a new stabilizer for the ethylene–norbornene copolymer [27].

The aim of the research was to determine the effect of the natural stabilizer naringenin on the physicochemical properties of epoxy resin composites reinforced with glass fibers. Moreover, the modification of commercial epoxy resin with flame retardants using naringenin was aimed at obtaining materials resistant to solar aging. The samples were subjected to controlled solar aging, and the stabilizing influence of the polyphenol was analyzed. In addition, antimicrobial tests were also performed, which allowed the evaluation of the properties of the samples against the microorganisms *E. coli* and *C. albicans*. Antibacterial properties make polymer compositions an attractive solution for rail car equipment in public transport and for other applications.

## 2. Results and Discussion

### 2.1. Microscopic Characteristics of Samples

**Figure 1:**
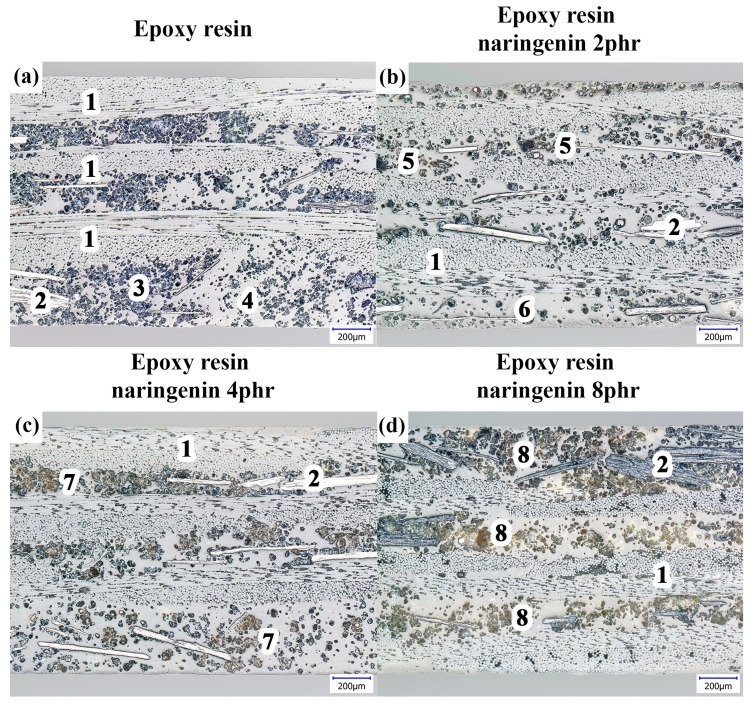
Cross-section optical images of (**a**) epoxy resin composite without additives, (**b**) composite containing 2 phr of naringenin, (**c**) composite containing 4 phr of naringenin, and (**d**) composite containing 8 phr of naringenin. 1—glass fabric; 2—graphite flakes; 3 and 4—APP powder uneven distribution; 5—naringenin inclusions; 6—APP deconcentration; 7—naringenin concentrations; 8—aggregations of APP and naringenin. All images obtained at magnification of 200×. phr means parts per hundred resin.

Microscopic observations were performed to determine the internal structure of the composites. Photographs of the cross-section microstructures of the samples are shown in Figure 1.

The image of the reference sample without naringenin (Figure 1a) shows the internal structure of the composite consisting of three layers of glass fabric (1) as a reinforcement and an epoxy matrix, in which the flame retardant additives graphite flakes (2) and ammonium polyphosphate (APP) in powder form (3) are dispersed. Despite the locally visible uneven distribution of these additives in the epoxy matrix (area (3) vs. area (4)), the separation of the reinforcement layers was maintained. Determined by counting the occupied surface area, the content of the flame retardant additive was estimated to be more than 20%, which is higher than the manufacturer’s declaration (<18%). Over the observed area, the microstructure was devoid of visible porosity and discontinuities. Figure 1b shows the microstructure of the sample with the 2 phr naringenin additive. The observed cross-section of the sample contained a noticeably lower content of the flame retardant additive throughout the matrix compared with the reference sample (Figure 1a). Areas of its uneven distribution were evident: numerous thickenings (5) and deconcentrations (6) (Figure 1b).

In some compaction areas (5), naringenin in the form of light-colored particles could be seen. These naringenin particles were well integrated inside the matrix of the composite. A further increase in the content of naringenin resulted in its more pronounced presence in the microstructures. In the case of sample containing 4 phr of naringenin (Figure 1c), a yellow-brown color area (7) of naringenin concentration could be distinguished. Their presence was also clearly visible in the case of the sample containing 8% naringenin (Figure 1d), where numerous sites of aggregation of naringenin with the flame retardant additive (8) could be seen. However, despite the cumulatively significant (>30%) amount of additives introduced into the matrix of the composite, no significant perturbation of the alignment of its reinforcement layers or increased matrix defectiveness was observed.

### 2.2. Determination of the Physicochemical Properties of Epoxy Resin Compositions

The effect of naringenin concentration on the density of the composites was analyzed. The density values of the samples were as follows: composite epoxy resin/naringenin 2 phr d = 1.65 g/cm^3^, epoxy resin/naringenin 4 phr d = 1.64 g/cm^3^, and epoxy resin/naringenin 8 phr d = 1.63 g/cm^3^. No relationship was found between the increase in naringenin concentration and the change in material density.

**Figure 2:**
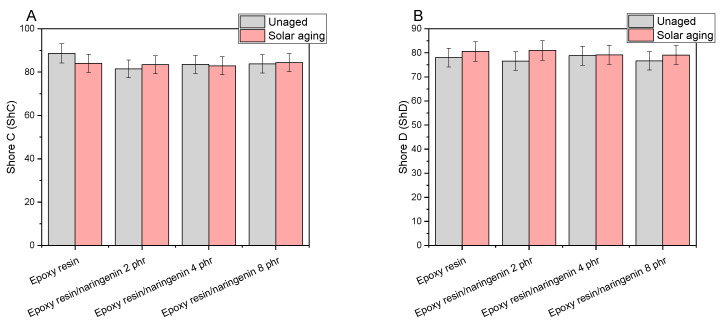
Hardness of epoxy resin compositions with naringenin before and after solar aging: (**A**) Shore C and (**B**) Shore D hardness scale.

Figure 2 summarizes the hardness values of epoxy resin with naringenin materials. The hardness of the epoxy resin reference sample was 88.6 ShC and 78.0 ShD. The addition of naringenin slightly changed the hardness of the composition. The hardness of the samples with naringenin measured on the Shore C scale was slightly lower than the value of the reference sample and fell within the range of 81.5–83.8 ShC. The hardness values determined on the D scale were identical for the reference sample and the samples with naringenin (76.6–78.8 ShD). No tendency of changes in hardness with increasing naringenin concentration was observed (the difference in values was ±2 ShC and ShD). The addition of naringenin in the amount of 2–8 phr did not significantly change the hardness of the epoxy resin composition. Solar aging did not cause any significant changes in the hardness of the composites. The hardness values of the samples after aging were slightly higher (1–5 Shore D and C units), except for the epoxy resin/naringenin 4 phr sample measured on the Shore C scale (before aging, 83.5 ShC; after aging, 82.9 ShC). Slight changes in the hardness value of the resin–naringenin composition may prove that the materials did not lose their resistance to local plastic deformation of the samples and that the functional properties were maintained during the given aging time. Similar results were obtained for the composition of epoxy resin with glass fibers, starch, and quercetin. The addition of biofiller and polyphenol did not negatively affect the hardness of the materials or their resistance to solar aging [25].

**Figure 3:**
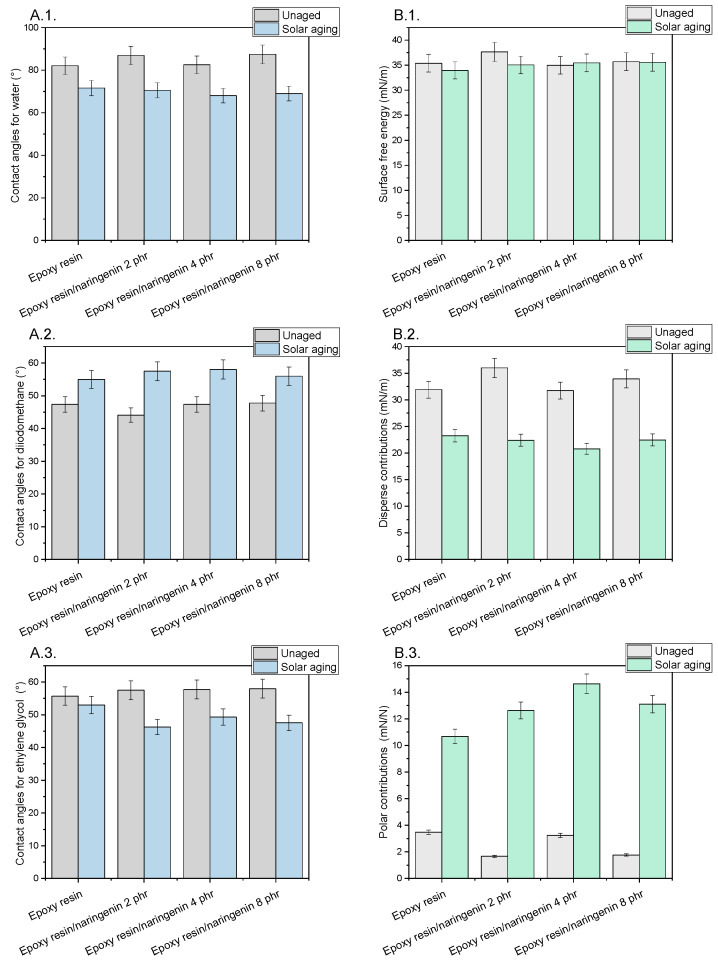
(**A.1**–**A.3**) Contact angles (°) before and after solar aging for water, diiodomethane, and ethylene glycol. (**B.1**–**B.3**) Surface free energy and its components (disperse and polar contributions) (mN/m) before and after solar aging.

Figure 3A.1–A.3 reviews the measurement results of the contact angles. The energy of the surface and its components (Figure 3B.1–B.3) of the epoxy resin composition before and after solar aging were calculated from the contact angles. The reference sample and compositions containing naringenin had similar surface properties. The contact angles measured for the three measuring liquids (water, diiodomethane, and ethylene glycol) were identical. The results for the surface energy and its components were also similar. The energy of the surface of the standard sample was 35.39 mN/m, while the samples with naringenin were 34–37 mN/m. All samples showed a small value of the polar component (1.67–3.47 mN/m). The dispersion component was 31–72–35.99 mN/m. No correlation was observed between the increase in the concentration of naringenin in the compositions and the change in the surface energy of the samples. Solar aging did not cause pronounced changes in the total surface energy but significantly changed the share of individual components, with a decrease in the value of the dispersive component and an increase in the value of the polar component found. The increase in the polar component of the surface energy of the polymer samples corresponded to the increase in their hydrophilic character, as well as the aging and degradation processes that occurred on the surface of the materials. A similar increase in the value of the polar component after solar aging of samples based on epoxy resin with biofiller and quercetin has also been described in the literature [25]. During controlled aging, functional groups related to their degradation reactions were generated on the surface of polymers, which results in the appearance of specific functional groups, including hydroxyl groups. The presence of OH groups caused an increase in the polar component and confirmed the start of degradation processes on the surface of the epoxy resin composition.

**Figure 4:**
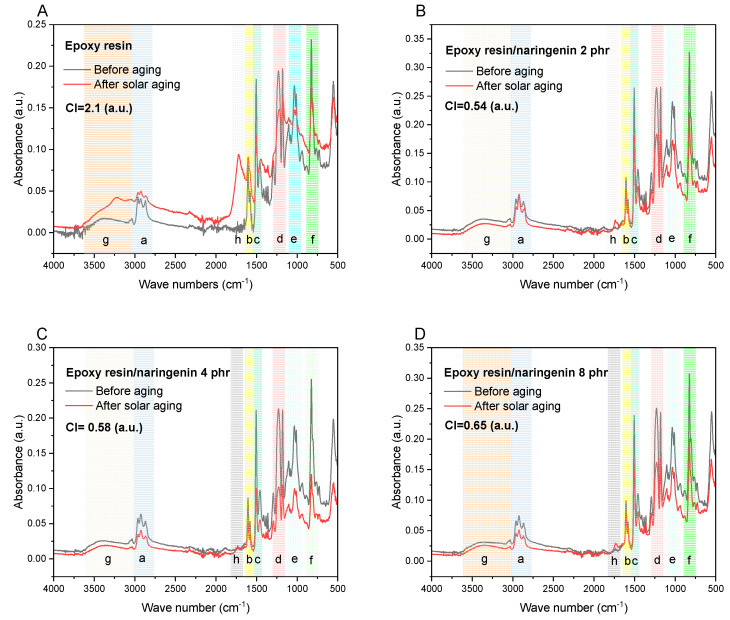
FTIR spectra of epoxy resin sample (**A**) and materials with naringenin (**B**–**D**) before and after solar aging. CI means carbonyl index.

The next step was the analysis of the FTIR spectra before and after solar aging (Figure 4). All FTIR spectra recorded for the composition with naringenin were reproducible, which proved the good homogeneity of the materials produced. Small differences in the intensity of the peaks could be related to the uneven distribution of the components contained in the composition with the resin (the samples showed fragments containing, e.g., a greater amount of flame retardants), the presence of fiberglass fabric on the surface of the samples, or the roughness of the analyzed area of the materials. The structure of the epoxy resin was not specified by the manufacturer. However, characteristic functional groups were recorded in the FTIR spectra, such as a triple band visible at 3000–2800 cm^−1^ (marked a) corresponding to -CH stretching, a band around 1600 cm^−1^ (marked b) relating to the characteristic of C=C stretching of aromatic compounds, a band around 1500 cm^−1^ (marked c) associated with stretching vibrations in the aromatic ring, a band around 1250 cm^−1^ (marked d) resulting from the presence of ester groups and/or oxirane rings specific to epoxy materials, a double signal in the range 1000–1050 cm^−1^ (marked e) coming from -CH_2_-OH groups, and also an intense band around 820 cm^−1^ (marked f) associated with the presence of C-C stretching vibrations of the epoxy ring [25].

In the FTIR spectra after solar aging, the appearance of additional hydroxyl groups (in the range of 3500–3000 cm^−1^, marked g) was observed, corresponding to the change in polarity of the samples, including surface energy parameters. Additionally, the presence of ketone groups at a wavenumber of about 1700 cm^−1^ (marked h) was observed, allowing the calculation of carbonyl indexes. The carbonyl index (CI) values were 0.54 (-) for the resin composition with 2 phr naringenin, 0.58 for the epoxy resin/naringenin 4 phr sample, and 0.65 (-) for the material containing 8 phr naringenin. The progress of degradation was comparable in all compositions; however, with the increase in polyphenol concentration, a slight increase in the value of carbonyl indexes was found, which may have been related to the oxidation processes of the natural additive. The carbonyl index value of the reference resin sample was 2.1 (-) and was approximately four times higher than the index values of samples containing naringenin. This indicated greater structural changes in the reference resin sample after solar aging, as well as the stabilizing effect of the natural additive.

Precise determination of the aging mechanisms of both polymeric materials and the action of natural stabilizers was difficult due to radical reactions and the stability of the formed radicals during subsequent stages of compound decomposition. During aging of polymer compositions containing naringenin as a natural stabilizer, the polyphenol was likely to undergo radical reactions. Stabilization of the ethylene–norbornene copolymer by using naringenin during UV aging has been proposed as possible radical reactions [27]. The scientific literature requires additional data on the behavior of naringenin itself during UV aging. However, such studies may be difficult due to the viability of the radical forms of the compound.

**Figure 5:**
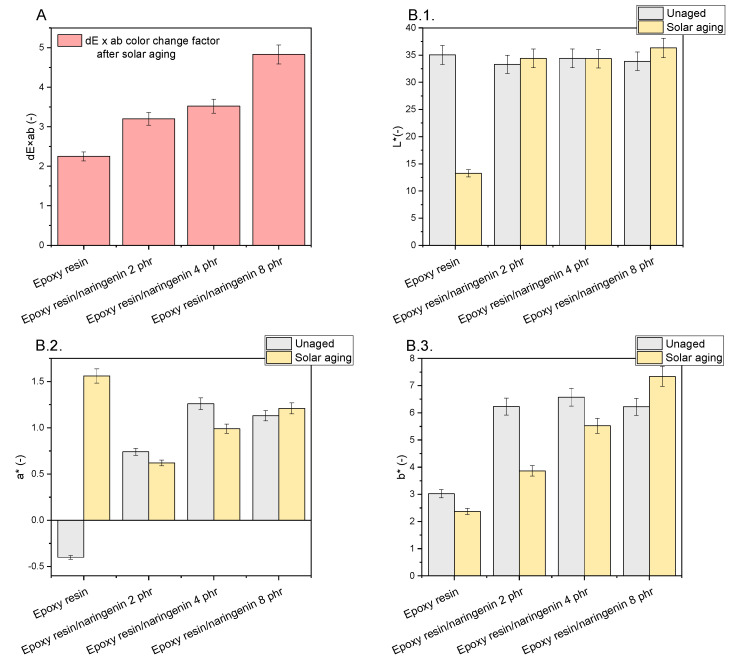
(**A**) Change in color dE × ab after solar aging and (**B.1**–**B.3**) parameters of the determination of the color of samples with naringenin.

The color analysis of the samples before and after aging was performed in the CIE-Lab color space (Figure 5). On the basis of spectrophotometric analysis, three color parameters were determined for the composites: L *, lightness index; a *, axis with positive values being red and negative values being green; and b *, axis with positive values being yellow and negative values being blue. For the reference resin sample, the parameters for determining the color before aging were as follows: parameter L = 35.05 (-), a * = 0.41 (-), and b * = 3.02 (-). In the case of compositions containing naringenin at a concentration of 2–8 phr, the samples were characterized by a comparable lightness factor L, and its value was 33–34 (-). The a * parameter of the materials with naringenin at various concentrations ranged from 0.74 to 1.26 (-), and the b * parameter ranged from 6.22 to 6.57 (-). The addition of white naringenin caused a slight lightening of the samples, confirmed by a slightly lower value of the lightness parameter L, compared with the standard resin sample.

The color change factor dE × ab after solar aging for epoxy resin with 2 phr of naringenin was 3.20 (-), which statistically meant that the difference in the color of the samples before and after aging was noticed by the average observer (2 < dE × ab < 3.5—the difference is noticed by the average observer). Samples with a higher concentration of naringenin were characterized by a higher value of the dE × ab coefficient, which was 3.52 (-) for epoxy resin/naringenin 4 phr and 4.83 (-) for epoxy resin/naringenin 8 phr. In the range of 3.5 < dE × ab < 5, the observer notices a clear difference in colors. As the concentration of naringenin increased, a greater change in the color of the samples was observed. A more pronounced color change in materials with naringenin can be caused by oxidation reactions of the polyphenol, causing its color to change. The reference resin showed the smallest color change. Naringenin oxidation may have caused a more pronounced color change in the samples.

**Figure 6:**
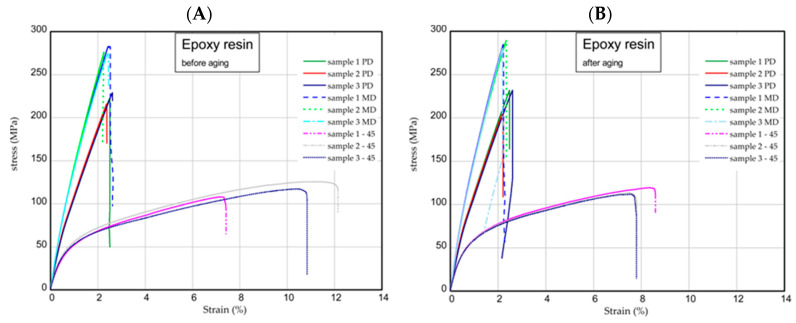
Curves of tension for pure resin epoxy before aging (**A**) and after aging (**B**).

**Figure 7:**
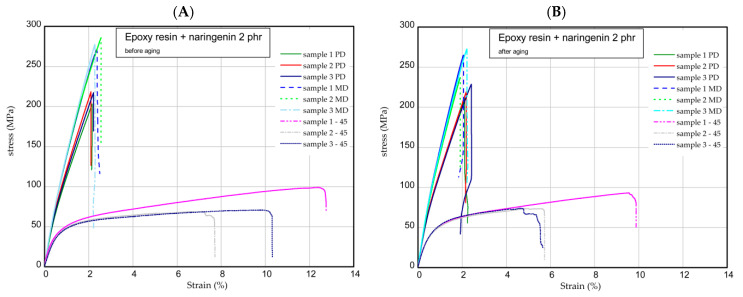
Curves of tension for pure resin epoxy/naringenin 2 phr before aging (**A**) and after aging (**B**).

**Figure 8:**
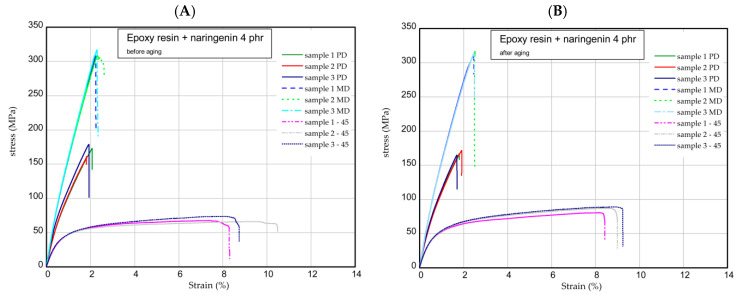
Curves of tension for pure resin epoxy/naringenin 4 phr before aging (**A**) and after aging (**B**).

**Figure 9:**
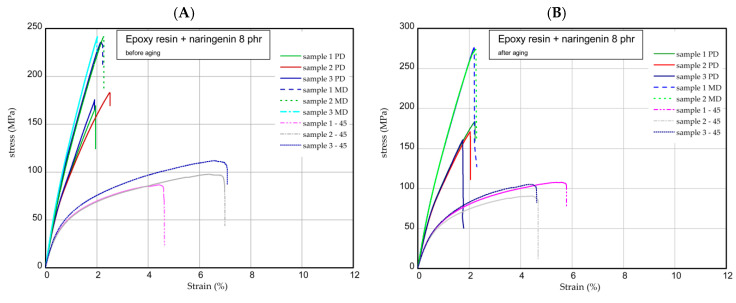
Curves of tension for pure resin epoxy/naringenin 8 before aging (**A**) and after aging (**B**).

The next step was the one-directional tensile test. This section shows the results of tensile tests of samples examined before and after the aging process. Tension tests were performed for three samples taken in three directions. Figure 6 presents the strain expressed in % vs. nominal stress in MPa (tensile forces referred to initial cross-section area) for pure epoxy resin. The maximum stress and Young’s modulus for MD samples were approximately 277 MPa and 17 GPa, respectively. In the case of PD samples, these values were registered lower by 10–15% than in the case of parameters obtained for MD samples. However, of the samples, 45 provided meaningfully smaller values (circa two times). Furthermore, the mechanical parameters remained stable after the UV process (Figure 6B). Characteristic parameters of all samples were collected in Table 1 and Table 2. Analyzing the next results (Figure 7, Figure 8 and Figure 9), in general, the increase in naringenin in the samples caused the growth of the strength (peaks were noticed for the samples with naringenin 4 phr) but only for the MD samples. Furthermore, the maximum stresses in PD samples with increasing naringenin content slightly dropped (see Figure 9 and the results inserted in Table 1 and Table 2). Based on present results, the influence of the aging process for the analyzed samples was not significant.

### 2.3. Determination of Microbiological Properties of Samples

**Figure 10:**
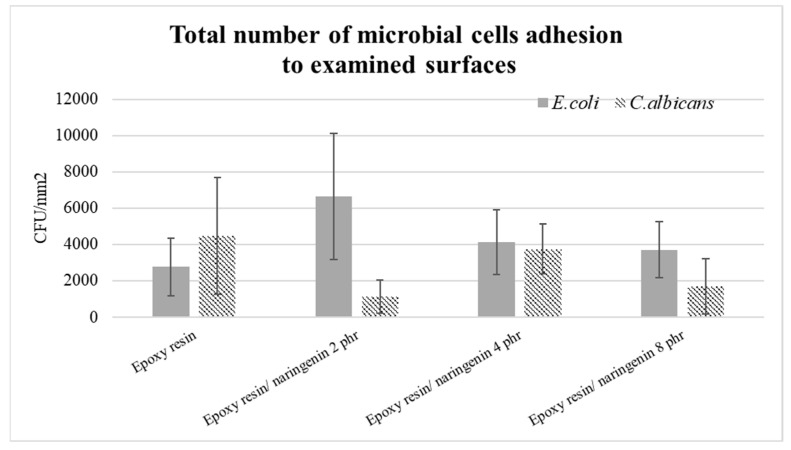
Potential of the inhibition of the microbial biofilm formation.

The last stage was the microbiological analysis of the samples. The results of the examination of the potential of inhibition of the microbial biofilm formation are presented in Figure 10. The total number of adhered *E.coli* cells to samples with embedded naringenin was higher than that for the reference sample; no effect of the addition of this substance on the bacteriostatic behavior of the examined materials was observed. However, in the case of inhibition of the adhesion of yeast cells, a positive effect was visible, i.e., a reduction in the total number of adhered *C. albicans* cells on the surfaces of the studied samples.

**Figure 11:**
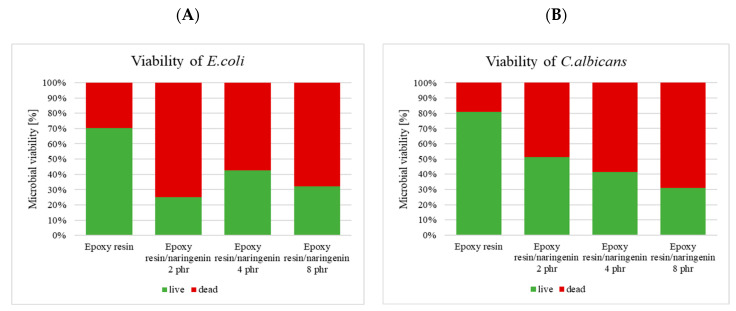
Viability of the microbial cells in contact with the examined surfaces of the samples. (**A**)—viability of the *E. coli*, (**B**)—viability of *C. albicans*.

In terms of the examination of viability of the microbial cells in contact with the examined surfaces, a decrease in the number of live cells in comparison with the control, undoped sample was observed, both in terms of bacteria (Figure 11A) and in terms of yeasts (Figure 11B). All samples examined exhibited good antimicrobial properties for both types of microorganisms.

## 3. Materials and Methods

### 3.1. Preparation of Composites Based on Epoxy Resin and Naringenin

Polymer compositions were made on the basis of an epoxy resin containing flame retardants (trade name NEMresin 1011; New Era Materials, Modlniczka, Poland). The mixture of resin and flame retardants had the following composition: approx. 73 wt.% pure epoxy resin, <18 wt.% poly(ammonium phosphate), and approx. 9 wt.% graphite. The resin belonged to the category of snap-cure resins, which meant that it was in the form of a powder at room temperature and cross-links under the influence of high temperature (above 100 °C). Naringenin (natural from Citrus paradisi Macf., 98%, Sigma Aldrich, Shanghai, China) was added to the base resin mixture in amounts of 2, 4, and 8 phr (phr means parts per hundred resin). The resin was mixed with naringenin, and the obtained powders were homogenized with a mortar and sieved. The samples of the polymer compositions consisted of alternating layers of resin and fiberglass fabric (RXT 350 g/cm^2^, Rymatex, Rymanów, Poland). Three layers of fiberglass fabric and three layers of epoxy resin were used, with the amount of resin kept at 450 g/cm^2^. The steel mold was covered with a silicone foil, on which a layer of fiberglass fabric was applied, followed by a mixture of resin and polyphenol (alternating three layers). The plate-like samples of polymer compositions were formed in two steps in a heated hydraulic press. The first step involved plasticizing the composition at 90 °C for 5 min. Then the samples were formed at the temperature of 130 °C, a pressure of 1 MPa, and a time of 8 min. After completion of the molding, the plate-like compositions were hardened in a laboratory oven at 136 °C for 45 min.

### 3.2. Microstructure Observations

The internal microstructures of the composites were investigated using the Keyence VHX-950F optical microscope (Keyence Corporation, Osaka, Japan) equipped with a VH-Z100R lens and proprietary image analysis software (Keyence System v3.2.0.121). Observations were conducted in cross-sections of the samples perpendicular to the plane of the reinforcing fabric. A hybrid lighting mode (bright and dark field) at 200× magnification was used.

### 3.3. Solar Aging

An Atlas SC 340 MHG Solar Simulator (AMETEK Inc., Berwyn, IL, USA) climate chamber equipped with a 2500 W MHG lamp was used for controlled solar aging. The rare earth halogen lamp provided a unique range of solar radiation (UV, Vis, and IR). The irradiance was 1200 W/m^2^ at 100% lamp power. Aging in the temperature of 70 °C lasted 800 h.

### 3.4. Determination of Sample Density

The density of the samples was determined using a set for determining the density of solids and liquids compatible with a laboratory balance from Radwag (Radom, Poland). The device enabled the determination of the density of solids and liquids. The process of density determination was fully automatic, i.e., the balance operator was limited to placing samples on the pan of the set. The sample density was determined in 96% ethanol, at a temperature of 21.7 °C. Before starting the measurements, three 1 × 1 cm sized samples weighing about 0.3 g were cut from the plates. The density measurement consisted of weighing the sample in air (on the upper pan of the set) and weighing the same sample in ethanol (on the lower pan of the set). The density result was automatically displayed on the balance display after the sample mass in the liquid was entered.

### 3.5. Determination of Contact Angles and Surface Free Energy

The OEC 15EC goniometer (DataPhysics Instruments GmbH, Filderstadt, Germany) was used to measure the surface free energy. The determination of the surface energy was performed on the basis of the measurement of the contact angle for liquids with different polarity: distilled water and diiodomethane. The free surface energy was calculated by the Owens, Wendt, Rabel, and Kaelble (OWRK) method. The SCA 20 software was used for the calculations. Polar and disperse contributions to surface energy and surface tension were combined by forming the sum of both parts, leading to Equations (1) and (2):

(1) $$\sigma_{l}=\sigma_{l}^{d}+\sigma_{l}^{p}$$

(2) $$\sigma_{S}=\sigma_{S}^{d}+\sigma_{S}^{p}$$

where $\sigma_{l}^{d}$ and $\sigma_{l}^{p}$ describe the disperse and polar parts of the liquid, while $\sigma_{s}^{d}$ and $\sigma_{s}^{p}$ stand for the respective contributions of the solid.

### 3.6. Fourier Transform Infrared (FTIR) Spectroscopy

A Thermo Scientific Nicolet 6700 FT-IR spectrometer (Thermo Fisher Scientific, Waltham, MA, USA) equipped with a Smart Orbit ATR diamond accessory was used for the measurements. Measurements were performed in the wavenumber range of 4000 to 400 cm^−1^ (64 scans, absorption mode). The samples were placed at the exit of the infrared radiation beams. As a result of the research, the vibrational spectra were obtained, the analysis of which allowed the determination of the functional groups with which the radiation interacted.

On the basis of the FTIR spectra, the carbonyl index (CI) was calculated, which informed the number of carbonyl groups formed during the aging process of the polymer compositions. The following Equation (3) was used for the calculations:

(3) $$CI=\frac{I_{C=O}}{I_{C-H}}$$

where *I_C_*_= *O*_ is the intensity of the peak corresponding to carbonyl groups C=O (~1700 cm^−1^) [-], and *I_C–H_* is the intensity of the peak corresponding to the aliphatic carbon C-H chains (~2800 cm^−1^) [-].

### 3.7. Determination of the Hardness of Polymer Compositions

Hardness measurements were performed using the Shore durometer type C and D. The determination consisted of measuring the depth of an indentation in the polymeric material created by a given force on a standardized presser foot.

### 3.8. Color Change Analysis

A CM-3600d spectrophotometer (Konica Minolta Sensing, Osaka, Japan) was used for color testing. Color measurements were performed before and after solar aging to determine the color change in the polymeric compositions. The result of the determination was the color as described in the CIE-Lab space and the color in a system of three coordinates: L, a, and b, where L is the lightness parameter (a maximum value of 100, representing a perfectly reflecting diffuser, and a minimum value of zero representing the color black), a is the axis of red–green, and b is the axis of yellow–blue. The a and b axes had no specific numerical limits. The change in color, dE × ab, was calculated according to Equation (4).

(4) $$dE\times ab=\sqrt{{(∆a}^{2})+\left({∆b}^{2}\right)+\left({∆L}^{2}\right)}$$

### 3.9. One-Directional Tensile Test

**Figure 12:**
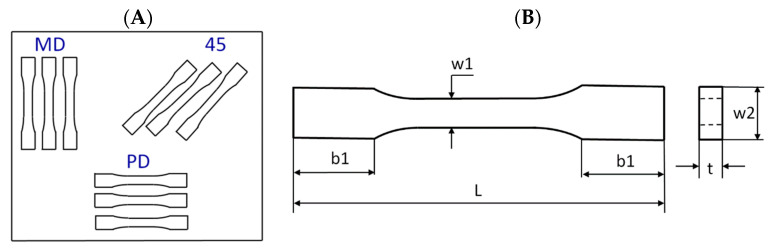
Scheme of taking samples (**A**) and drawing of sample (**B**).

Tensile tests were carried out according to the UNE EN ISO 527-1:2020-01 standard [28] using an INSTRON test machine. According to the standard, the constant speed of the moving traverse was assumed to be equal to 2 mm/min. As a result of the tests, the diagrams of force versus elongation were obtained. The samples were cut out from the plate according to scheme shown in Figure 12A (MD, main direction; PD, perpendicular direction; and 45, an angle orientated to the plate edges). The dimensions of the samples were based on a standard: L = 170 mm, b1 = 40 mm, w1 = 10 mm, w2 = 20 mm, and mean thickness t = 3 mm (Figure 12B). The Young’s modulus of the composite was determined by using an extensometer with a gauge length of 50 mm.

### 3.10. Microbiological Assay

The samples were subjected to investigations of their antimicrobial properties. Two parameters were taken into account—the ability of the sample surfaces to inhibit the formation of the microbial biofilm and the ability to counteract the viability of the microorganisms that were in contact with the examined samples’ surfaces. All tests were carried out for two microbial strains: *Escherichia coli* (ATCC, DH5α strain) and *Candida albicans* (ATCC 10231).

The test involving the evaluation of microbial adhesion inhibition by the examined surfaces was performed by means of fluorescence microscopy. Clean and disinfected samples were incubated for 24 h in a liquid medium at 37 °C (Luria Bertani in the case of *E. coli* and YPG for *C. albicans*) with grown microorganisms. The media were inoculated using approximately 2 × 10^3^ cells. After this time, the samples were removed from the media and gently rinsed with deionized water to remove the unadhered microbial cells from the samples’ surfaces. Then, the microbial cells were stained for the visualization of cells that had remained attached to the examined surfaces. For that purpose, bis-benzidine (live cell visualization) and propidium iodide (dead cells) were used: 5 μL of stock solution of each dye (100 μg/mL) was applied, and after 10 min of incubation of samples in room temperature in the dark, visualization of the surfaces was initialized. Microbial cells were detected using an Olympus GX71 fluorescent microscope and a CCD camera (DC73), at 10 different randomly selected spots, and the procedure was repeated for three different samples. Image acquisition was performed with the help of analySIS DOCU software (Olympus Soft Imaging Solution Stream Essentials 1.9). The count of the total number of cells adhered to the surfaces was performed with ImageJ freeware software.

The second test involved the examination of the antimicrobial potential of the surfaces by calculating the amounts of viability of the cells after contact with the examined materials. Each surface was treated with the microbial inoculum that was in the logarithmic growth phase. After 60 min of incubation at 37 °C (i.e., contact of the inoculum with the surfaces), it was collected from the surfaces and stained with the Viability/Cytotoxicity Assay Kit for Live and Dead Bacteria (Biotium, Fremont, CA, USA), which was composed of DMAO, 5 mM solution (staining of both live and dead cells), and EthD-III, 2 mM solution (staining only dead bacteria with damaged cell walls). Calculations of the percentages of live and dead cells were performed using the flow cytometry technique, with the C6 Flow Cytometer (Becton Dickinson, Franklin Lakes, NJ, USA). As a reference, two samples were used: a positive control (low microbial viability, microbial inoculum with 98% methanol added) and a positive control (high microbial viability, pure inoculum).

## 4. Conclusions

- Innovative solutions such as replacing synthetic stabilizers with compounds of natural origin are expected in current polymer technologies. An important industry in which epoxy materials are used is the railway industry, where seats made of epoxy resins and glass fibers are used. The solutions proposed in this work increased the environmental aspect of epoxy materials because of the application of a natural stabilizer from the group of polyphenols naringenin. Naringenin provided compositions with adequate resistance to solar aging associated with the use of railway wagons. Replacing commercial stabilizers with naringenin obtained from citrus waste may have a positive impact on the economic properties of epoxy compositions by reducing its price. Furthermore, the prototypes of seat elements obtained showed antimicrobial properties, which is an important and beneficial feature in public transport by potentially reducing microbiological threats to passengers.
- The addition of naringenin did not significantly affect the functional properties (hardness) and surface properties (surface energy and its parameters) of the epoxy materials. In addition, no effect of increasing the concentration of naringenin on the investigated properties of the samples before aging was found.
- Solar aging lasting 800 h did not cause significant changes in the hardness (82.9–88.6 Shore C and 76.1–81 Shore D) of the samples, which meant that they retained their resistance to local plastic deformation. Furthermore, the mechanical parameters also remained stable after the aging process.
- The surface energy of the materials also did not decrease after controlled aging, but the polar component corresponding to the change in polarity of the samples increased (about three to eight times), thus indicating the beginning of the degradation process of the resin composition. The carbonyl indexes of naringenin-containing materials, calculated from FTIR spectra, were about four times lower than those for the reference sample, which meant that naringenin stabilized the polymer composites. Materials containing naringenin were characterized by a greater color change (about 1.5–2 times) than the reference sample, which was associated with a change in the color of the polyphenol during its oxidation.
- Naringenin can be used as a potential antimicrobial agent in epoxy resin composites. In the test of microbial cell viability in contact with composite surfaces, a decrease in the number of viable bacterial and yeast cells was found compared with the reference sample.

## Figures and Tables

**Table 1:** Main values of Young’s modulus in (GPa).

Sample	Before Aging			After Aging		
	PD	MD	45	PD	MD	45
**Epoxy resin**	14.36 ± 0.40	17.31 ± 0.20	9.34 ± 0.65	14.78 ± 0.45	18.43 ± 0.24	10.39 ± 0.18
**Epoxy resin/naringenin 2 phr**	15.53 ± 0.52	16.58 ± 0.30	8.72 ± 0.49	16.23 ± 0.06	17.83 ± 0.44	9.24 ± 0.07
**Epoxy resin/naringenin 4 phr**	15.05 ± 1.46	18.97 ± 0.38	8.19 ± 0.31	15.49 ± 0.66	18.55 ± 0.08	9.22 ± 0.27
**Epoxy resin/naringenin 8 phr**	14.34 ± 0.08	16.28 ± 1.09	9.20 ± 0.41	15.43 ± 0.13	18.13 ± 0.01	9.63 ± 0.41

**Table 2:** Main values of maximum stress in (MPa).

Sample	Before Aging			After Aging		
	PD	MD	45	PD	MD	45
**Epoxy resin**	223.6 ± 6.7	277.7 ± 4.6	116.9 ± 8.9	223.8 ± 14.2	284.9 ± 4.7	114.9 ± 4.0
**Epoxy resin/naringenin 2 phr**	212.9 ± 8.5	277.9 ± 7.6	79.5 ± 16.8	219.9 ± 8.6	258.1 ± 18.8	80.3 ± 11.2
**Epoxy resin/naringenin 4 phr**	171.1 ± 8.9	311.4 ± 4.8	69.2 ± 4.1	167.8 ± 3.4	313.6 ± 3.7	85.6 ± 4.5
**Epoxy resin/naringenin 8 phr**	176.06 ± 6.9	239.5 ± 3.4	98.8 ± 12.7	171.8 ± 11.4	274.8 ± 1.3	101.0 ± 9.3

## Data Availability

Data are contained within the article.
